# Phase separation of DDX21 promotes colorectal cancer metastasis via MCM5-dependent EMT pathway

**DOI:** 10.1038/s41388-023-02687-6

**Published:** 2023-04-07

**Authors:** Huabin Gao, Huiting Wei, Yang Yang, Hui Li, Jiangtao Liang, Jiecheng Ye, Fenfen Zhang, Liyuan Wang, Huijuan Shi, Jia Wang, Anjia Han

**Affiliations:** 1 Department of Pathology, the First Affiliated Hospital, Sun Yat-Sen University, Guangzhou, 510080 China; 2 GMU-GIBH Joint School of Life Sciences, The Guangdong-Hong Kong-Macau Joint Laboratory for Cell Fate Regulation and Diseases, Guangzhou Medical University, Guangzhou, 511436 China

**Keywords:** Colorectal cancer, Biomarkers

## Abstract

RNA binding proteins (RBPs) contributes to cancer progression, but the underlying mechanism reminds unclear. Here, we find that DDX21, a representative RBP, is highly expressed in colorectal cancer (CRC), which leads to CRC cell migration and invasion in vitro, and CRC to liver metastasis and lung metastasis in vivo. This effect of DDX21 on CRC metastasis is correlated to the activation of Epithelial-mesenchymal transition (EMT) pathway. Moreover, we reveal that DDX21 protein is phase separated in vitro and in CRC cells, which controls CRC metastasis. Phase-separated DDX21 highly binds on *MCM5* gene locus, which is markedly reduced when phase separation is disrupted by mutations on its intrinsically disordered region (IDR). The impaired metastatic ability of CRC upon DDX21 loss is restored by ectopic expression of *MCM5*, indicating *MCM5* is a key downstream target of DDX21 for CRC metastasis. Furthermore, co-higher expressions of DDX21 and MCM5 is significantly correlated with poor survival outcomes of stage III and IV CRC patients, indicating the importance of this mechanism in CRC late and metastatic stage. Altogether, our results elucidate a new model of DDX21 in regulating CRC metastasis via phase separation.

## Introduction

The phenomenon of liquid-like phase separation is observed in numerous membrane-less organelles and proteins, which plays key roles in most kinds of biological processes [1–3]. Protein phase separation is mediated by weak multivalent interactions between nucleic acids and proteins, which usually occurs on the intrinsically disordered regions (IDRs) [4, 5]. Phase separation can regulate robust gene expression by driving the formation of super-enhancers [6, 7]. Moreover, phase-separated condensates can select targeting genes by including specific components, resulting in cell fate determination [8]. In addition, phase separation also participates in genome organizations [9, 10]. Aberrant phase separation caused by mutations on IDRs regulates carcinogenesis [11]. A leukemia specific fusion protein NUP98-HOXA9 by chromatin translocation drives aberrant phase separation and promotes cancer development [12]. AKAP95 protein condensates regulate splicing and tumorigenesis, and disabled phase separation by point mutation can abolish its effect [13]. These studies provide high correlation between phase separation and cancers, however, direct causal relationship remains to be elucidated.

RNA binding proteins (RBPs) are one kind of characteristic protein types for phase separation. RBPs have both RNA recognition motif (RRM) and IDR to interact with RNA, which facilitates and stabilizes their phase-separated condensates [14]. Phase separation of RBPs is often observed in RNA granules, RNA speckles and nucleoli, which deeply participates in RNA transcription, splicing and modifications [15, 16]. DNA mutations that occur on the IDRs of RBPs, such as FUS, Tau, TDP43 and hnRNPA1, often change their phase separation properties, leading to irreversible aggregation of amyloid-like fibers and neurodegenerative diseases [17]. RBPs are also abnormally expressed in cancer, which leads to much more malignance [18]. For instance, CELF1 can target *ETS2* gene, which results in the chemoresistance of CRC [19]. Moreover, DENR deficiency disrupts tumor growth by inhibits JAK translation and PD-L1 expression [20]. However, the deep-seated mechanism by which RBPs contribute to carcinogenesis is not fully understand.

As the largest RNA helicase family, the members of the DEAD/H-BOX family are representative RBPs. There are 59 of DEAD/H-box helicases, comprising of 44 of DEAD-box (DDX) helicases and 15 of DEAH-box helicases [21], which are involved in most RNA physiological processes such as including RNA transcription, editing, splicing and transport [22, 23]. Aberrant expression of DDX family proteins play an important role in cancer progression, especially in CRC [24, 25], however, the inner molecular mechanism remains unclear. Here, we identified DDX21 as an oncogene for CRC progression. DDX21 forms phase-separated condensates with liquid-like behavior in CRC, which promotes CRC cell metastasis. This effect may partially attribute to the enhanced transcription of *MCM5*, a specific target of DDX21 phase-separated condensates. Overall, our findings provide a novel DDX21-phase separation axis for targeted therapeutic strategy of metastatic CRC.

## Results

### DDX21 promotes CRC metastasis both in vitro and in vivo

**Fig. 1:**
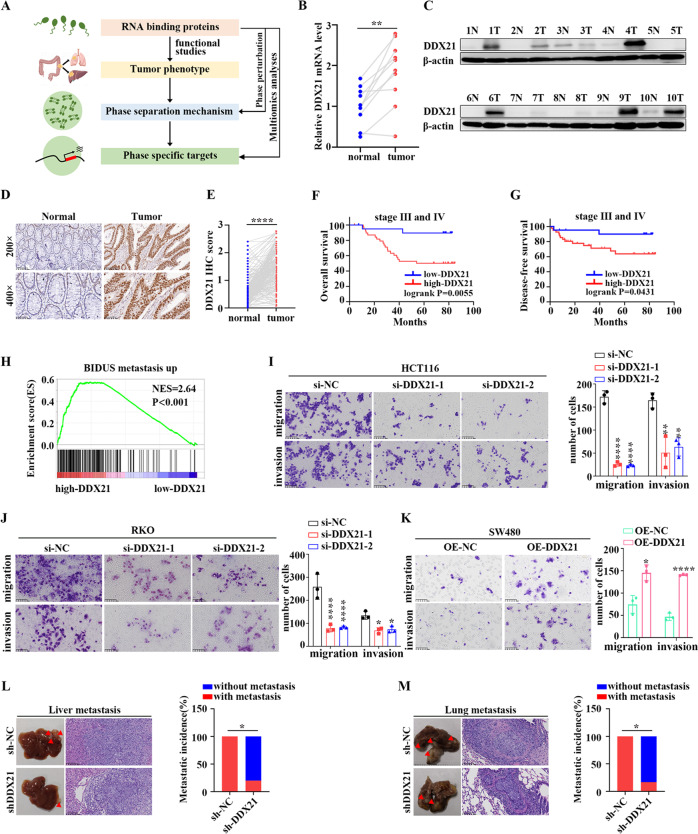
DDX21 promotes CRC metastasis both in vitro and in vivo. **A** Scheme of the research route and methods in this study. **B** qPCR results reveal that DDX21 are highly expressed in fresh CRC tissues than adjacent normal tissues in ten CRC patients. **P < 0.01. Results are from three biological replicates. **C** Western blots show that DDX21 proteins are highly expressed in CRC tissues than adjacent normal tissues in ten CRC patients. β-actin is used as a loading control. Two biological replicates are assayed for western blotting experiment. **D** Representative IHC images of DDX21 in CRC tissues and adjacent normal tissues. Dark brown stands for higher DDX21 expression while light blue means lower expression. **E** Quantitative analysis of IHC showing DDX21 is highly expressed in CRC tissues. ****P < 0.0001. Kaplan-Meier analysis of the OS rate (**F**) and DFS rate (**G**) in CRC patients of stage III and IV according to DDX21 expression. **H** GSEA analysis based on high-DDX21 group and low-DDX21 group in TCGA CRC cohort. The influence of DDX21 knockdown on HCT116 (**I**) or RKO (**J**) cell migration and invasion ability is evaluated by transwell assay. *P < 0.05, **P < 0.01, ****P < 0.0001. Results are from three biological replicates. **K** The influence of DDX21 overexpression on SW480 cell migration and invasion ability is evaluated by transwell assay. *P < 0.05, ****P < 0.0001. Results are from three biological replicates. Representative images of liver metastasis (**L**) and lung metastasis (**M**) derived from sh-NC and sh-DDX21 group of nude mice. Metastatic incidence of liver metastasis and lung metastasis is calculated. Scale bar stands for 100 μm length. *P < 0.05. The data in (**I**)–(**K**) are presented as the means ± SD.

To determine the significance of RBPs in cancer, we detected the role of representative RBP in CRC phenotype, followed by investigating the potential phase separation mechanism and identifying phase separation-specific targets (Fig. 1A). As DDX family is representative RBP family and plays an important role in cancer regulation [24], we selected the candidate protein from this family. DDX21 is outstanding by integrating the RNA-seq analyses from three public databases (Fig. S1A–C) [26–28]. We evaluated the expression difference of DDX21 between CRC and adjacent normal tissues by qPCR, western blots and IHC, respectively, and found DDX21 expression level is significantly higher in CRC tissue than that in adjacent normal tissues (Fig. 1B–E). In line with these, we observed protein levels of DDX21 in most of CRC cell lines, such as HCT116, LOVO and RKO, are significantly higher than those in normal colonic epithelial cell NCM460 except SW480 (Fig. S1D). Moreover, upregulation of DDX21 was correlated with poor Overall Survival (OS) rate and Disease-free Survival (DFS) in stage III and IV CRC patients (Fig. 1F, G), rather than among all stage or stage I and II (Fig. S1E–H), indicating DDX21 may have potential role in late stage CRC. Furthermore, gene set enrichment analysis (GSEA) of the expression profiles of CRC from the TCGA database revealed that high expression of DDX21 group was significantly enriched in “BIDUS metastasis up” gene set (Fig. 1H), demonstrating that DDX21 may contribute to CRC metastasis.

To verify the role of DDX21 in regulating CRC metastasis, firstly, we knocked down *DDX21* in HCT116 and RKO CRC cell lines that have higher endogenous DDX21 expression (Fig. S1I). Loss of DDX21 significantly reduced the CRC migration and invasion ability both in HCT116 and RKO cells (Fig. 1I, J). Secondly, we overexpressed *DDX21* in SW480 CRC cell line that has lower endogenous DDX21 expression (Fig. S1J). Results showed that ectopic expression of DDX21 significantly enhanced CRC migration and invasion ability in SW480 cells (Fig. 1K, S1K, L). Previous results showed that loss of DDX21 expression suppressed the CRC cell proliferation, cell cycle and tumor growth [29, 30], suggesting that DDX21 may play an important role in the tumorigenesis of CRC. We also validated the pro-proliferation effect of DDX21 in CRC (Fig. S1M–O). To further validate the pro-metastatic capacity of DDX21, thirdly, we transplanted HCT116 cells with knockdown of *DDX21* or control cells into BALB/c mice by intrasplenic injection and tail vein injection (Fig. S1P). The xenografts were harvested at 6–8 weeks after injection, and we found that most of the heterologous cells are accumulated at livers and lungs, while few are at hearts and soft tissues (Fig. 1L, M, S1Q, R). Notably, Loss of DDX21 remarkably suppressed CRC liver and lung metastasis as compared with control cells (Fig. 1L, M), supporting the role of DDX21 in facilitating CRC to liver metastasis and lung metastasis.

Taken together, these results demonstrate that DDX21 which is highly expressed in CRC promotes CRC metastasis.

### DDX21 enhances CRC metastasis through the EMT signaling pathway

**Fig. 2:**
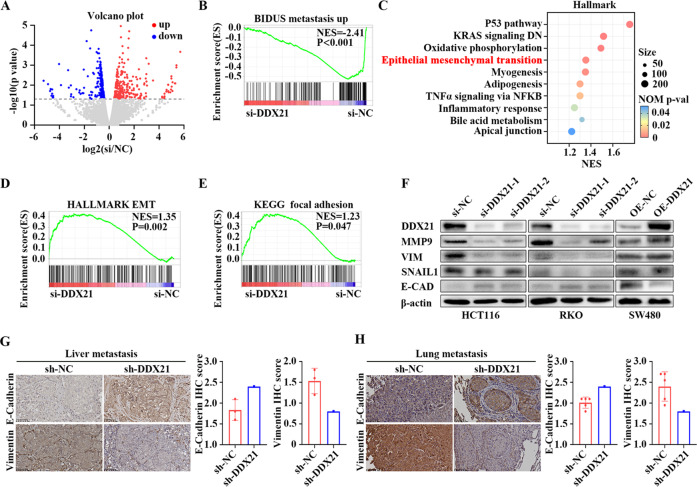
DDX21 enhances CRC metastasis through the EMT signaling pathway. **A** Volcano plot showing the distribution of differentially expressed genes (DEGs) between control and DDX21 knockdown cells. Results are from three biological replicates. Red dots represent upregulated genes and blue dots represent downregulated genes. **B** GSEA analysis showed that DDX21 significant associated with “BIDUS metastasis up” gene set. **C** Representative Hallmark gene set enriched in si-DDX21 vs si-NC group. EMT (Hallmark gene set) (**D**) and KEGG focal adhesion gene sets (**E**) enriched in si-DDX21 group vs si-NC group. **F** Western blots showing DDX21 protein expression is positively correlated to the expressions of the key components of EMT signaling pathway in CRC cells. β-actin is used as a loading control. Two biological replicates are assayed for western blotting experiment. E-cadherin and vimentin staining of metastatic tumors in the livers (**G**) and lungs (**H**) with HCT116-sh-DDX21 and HCT116-sh-NC cells. Scale bar stands for 50 μm length. The protein expression was determined by IHC score.

To elucidate the underlying mechanisms of DDX21 in CRC metastasis, we compared the transcriptomes of HCT116 cells with or without DDX21 loss. Results showed that loss of DDX21 induced significant transcription changes of considerable genes (Fig. 2A). GSEA analysis between the si-DDX21 and si-NC groups showed that DDX21 significant associated with “BIDUS metastasis up” gene set (Fig. 2B). Moreover, we noted that DDX21 were highly correlated with “Hallmark EMT” and “KEGG focal adhesion”. (Fig. 2C–E), which were consistent with the GSEA result based on TCGA CRC cohort (Fig S2A, B). We also examined the expressions of epithelial marker E-cadherin and mesenchymal markers vimentin, MMP9 and Snail both in DDX21 knockdown or overexpressed cells by western blots. The results showed that loss of DDX21 suppressed the expressions of mesenchymal markers but enhanced the expression of epithelial marker (Fig. 2F). In contrary, overexpression of DDX21 enhanced the levels of mesenchymal markers but reduced the level of epithelial marker (Fig. 2F). Third, IHC of E-cadherin and vimentin in liver metastatic tumor, lung metastatic tumor and soft tissue metastatic tumor models revealed that loss of DDX21 enhanced E-cadherin but reduced vimentin expression (Fig. 2G, H, S2C, D). All these results suggest that loss of DDX21 inhibits the transfer from epithelium to mesenchymal.

Taken together, these results suggest that DDX21 enhances CRC metastasis through EMT signaling pathway.

### DDX21 forms phase-separated condensates with liquid-like behavior in CRC cells

**Fig. 3:**
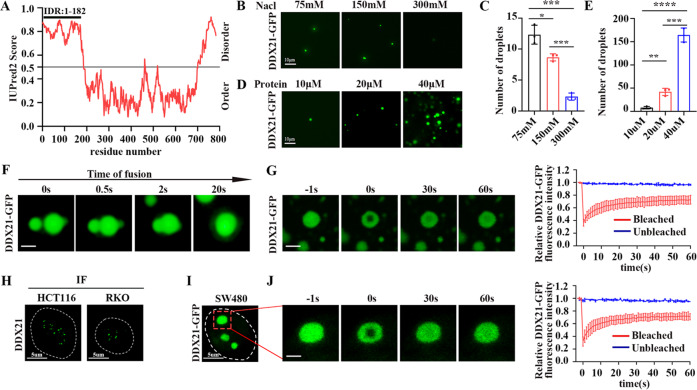
DDX21 forms phase-separated condensates with liquid-like behavior in CRC cells. **A** Graph of intrinsic disordered region (IDR) of DDX21 as calculated by the IUPred2 website (https://iupred2a.elte.hu/plot). IDR (1-182 amino acid) is indicated above the disorder score graph. **B** Representative images of droplet formation of 10 mM DDX21-GFP in droplet formation buffer with varying concentrations of salt and 10% PEG-8000. **C** Quantitative analysis of droplet formation of 10 mM DDX21-GFP in droplet formation buffer with varying concentrations of salt and 10% PEG-8000. The number of droplets decreases with increased salt concentration. *P < 0.05, ***P < 0.001. **D** Representative images of droplet formation of DDX21-GFP at the indicated protein concentrations in droplet formation buffer with 125 mM NaCl and 10% PEG. **E** Quantitative analysis of droplet formation of DDX21-GFP in droplet formation buffer with varying concentrations of protein. The number of droplets increases with increased protein concentration. **P < 0.01, ***P < 0.001, ****P < 0.0001. **F** Two DDX21 droplets can fuse into one larger droplet. Scale bar stands for 2 μm. **G** FRAP of DDX21-GFP droplets. Confocal images were taken at indicated time points relative to photo bleaching (0). Scale bar stands for 2 μm. **H** The in vivo phase-separated condensates of endogenous DDX21 protein are shown in HCT116 and RKO cells by IF. **I** The in vivo phase-separated condensates of exogenous DDX21-GFP protein are shown in SW480 cells. **J** FRAP of in vivo DDX21-GFP condensates in SW480 cells. Confocal images were taken at indicated time points relative to photo bleaching (0). Scale bar stands for 2 μm. The data in (**C**), (**E**), (**G**) and (**J**) are presented as the means ± SD.

Since RBPs are likely to be phase separated in biological processes [15], we asked whether DDX21 undergoes phase separation in CRC. DDX21 has a strong IDR at its N-terminal that includes 182 amino acids (Fig. 3A), indicating DDX21 has phase separation potential. In vitro droplet formation assay showed that DDX21 can form circle-like droplets in vitro under salt solution, and the number of droplets shifts towards lesser with increasing salt concentration (Fig. 3B, C). Moreover, these droplets are smaller at low protein concentration and shift towards bigger when protein concentration is increased, and the number of droplets is increased in higher protein concentration (Fig. 3D, E). Furthermore, two droplets can fuse into one larger droplet (Fig. 3F), and the fluorescence signal of droplets can fast recover after photobleaching (Fig. 3G), indicating that the inner of the droplets has liquid-like behavior. Furthermore, we detected DDX21 status in CRC cells. The endogenous DDX21 can aggregate into condensates in CRC cell lines by immunofluorescence (IF) (Fig. 3H). When we transfected the exogenous DDX21-GFP into SW480 CRC cell line that has low endogenous DDX21 expression, DDX21 can form larger condensates (Fig. 3I). Importantly, the GFP signal can fast recover after photobleaching (Fig. 3J), indicating the DDX21 condensates in CRC cells are liquid.

Taken together, these results demonstrate that DDX21 can be phase separated both in vitro and in CRC cells.

### Phase separation of DDX21 regulates CRC metastasis

**Fig. 4:**
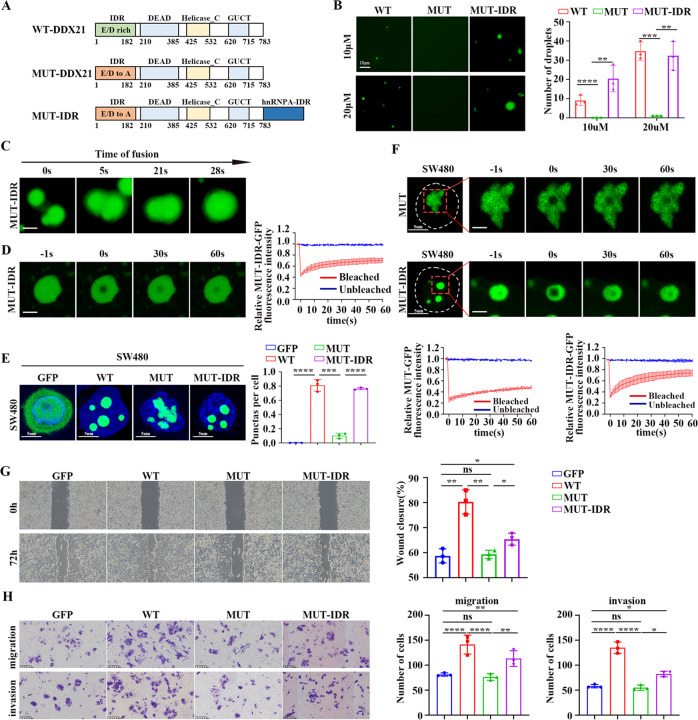
Phase separation of DDX21 regulates CRC metastasis. **A** Domain structure of WT-DDX21, MUT-DDX21 and MUT-IDR are showed. **B** Representative images and quantitative analysis of droplet formation of WT-DDX21, MUT-DDX21 and MUT-IDR at the indicated protein concentrations in droplet formation buffer with 125 mM NaCl and 10% PEG. Acidic mutations abolished droplet formation ability of DDX21 in vitro and hnRNPA1-IDR fusion recover the disabled droplet formation ability of the mutant. **P < 0.01, ***P < 0.001, ****P < 0.0001. Results are from three biological replicates. **C** Two MUT-IDR droplets can fuse into one larger droplet. Scale bar stands for 2 μm. **D** FRAP of MUT-IDR droplets. Confocal images were taken at indicated time points relative to photo bleaching (0). Scale bar stands for 2 μm. **E** Representative images and quantitative analysis of droplet formation of GFP, WT-DDX21, MUT-DDX21 and MUT-IDR in SW480 cells. Acidic mutations abolished droplet formation ability of DDX21 in SW480 cells and hnRNPA1-IDR fusion recover the disabled droplet formation ability of the mutant. ***P < 0.001, ****P < 0.0001. Results are from three biological replicates. **F** FRAP of MUT-DDX21 and MUT-DDX21-IDR droplets. Confocal images were taken at indicated time points relative to photo bleaching (0). Scale bar stands for 2 μm. **G** Wound healing assay is used to demonstrate the migrated ability of SW480-GFP, WT-DDX21, MUT-DDX21 and MUT-IDR cells. *P < 0.05, **P < 0.01. Results are from three biological replicates. **H** Migration and invasion ability of the above cells were analyzed by transwell assay. *P < 0.05, **P < 0.01, ***P < 0.001, ****P < 0.0001. Results are from three biological replicates. The data in (**B**), (**D**)–(**H**) are presented as the means ± SD.

To clarify the role of DDX21 phase separation on CRC metastasis, we designed DDX21 mutants to manipulate its phase separation ability. Since acidic mutation at IDR can change phase separation property of proteins [6, 10], we generated DDX21 mutant wherein all glutamic and aspartic acids in DDX21 N-terminal IDR were replaced with alanine (Fig. 4A, S3A). Moreover, we generated the rescued DDX21 mutant by fusing the IDR of hnRNPA1 (Mut-IDR) that is known to drive condensate formation (Fig. 4A) [14]. We observed that acidic mutations abolished droplet formation ability of DDX21 in vitro, resulting in diffuse status of DDX21 in salt solution (Fig. 4B). Importantly, the disabled droplet formation ability of the mutant can be recovered via hnRNPA1-IDR fusion (Fig. 4B,S3B). Moreover, like wild type (WT) DDX21, the droplets can fuse (Fig. 4C), and the fluorescence signal of Mut-IDR droplets can fast recover after photobleaching (Fig. 4D), indicating that the inner of the Mut-IDR droplets has liquid-like behavior.

Next, we transfected the GFP, WT-DDX21, acidic mutant DDX21 and Mut-IDR into SW480 cells, respectively (Fig. S3C). As expected, different DDX21 patterns display different phase separation capacity in CRC cells, in which WT and Mut-IDR DDX21 are liquidly phase separated, DDX21 mutant is poorly liquid, and GFP alone is diffuse (Fig. 4E). Also, FRAP assay show that the fluorescence signal of DDX21 mutant recover slowly after photobleaching while Mut-IDR droplets can fast recover after photobleaching in SW480 cells (Fig. 4F). Intriguingly, overexpression of WT-DDX21 robustly enhances CRC cell migration and invasion ability as compared with those of GFP only cells (Fig. 4G, H). Acidic mutations which disrupt DDX21 phase separation attenuate DDX21 effect on metastasis. The disabled metastatic function of DDX21 mutant is restored by IDR fusion which rescue DDX21 phase separation ability (Fig. 4G, H). Moreover, the same result of cell migration and invasion were observed by another CRC cell line HT29 (Fig. S3D, E). These results illustrate that disruption of DDX21 condensates suppresses CRC metastasis, which can be restored by condensate rescue via IDR fusion.

Taken together, these results demonstrate that DDX21 phase separation directly regulates CRC metastasis.

### *MCM5* is a direct target of DDX21 phase separation

**Fig. 5:**
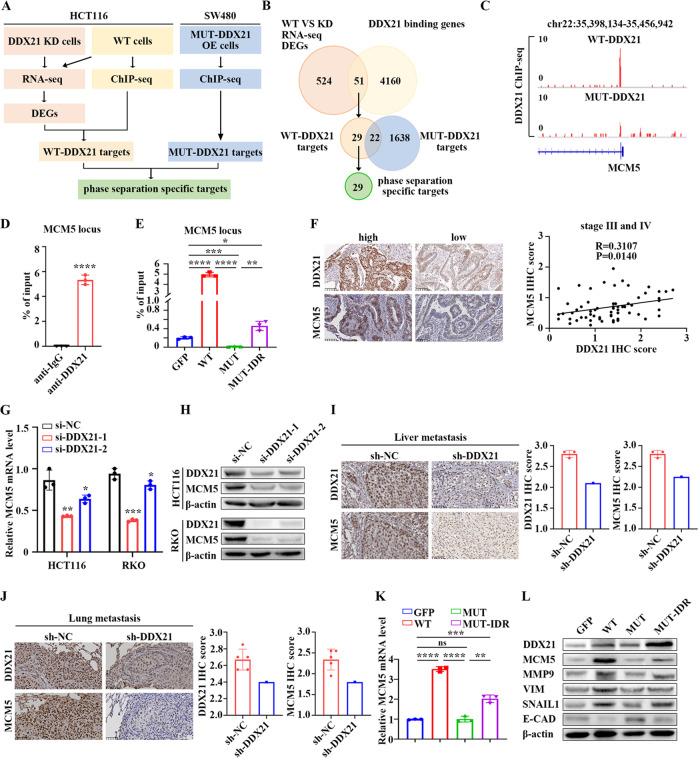
MCM5 is a direct target of DDX21 phase separation. **A** Schematic diagram showing the screening strategy for specific targets of DDX21 phase separation. **B** Venn diagram showing 29 candidates are identified as the targets of DDX21 phase separation in CRC. **C** The gene region of MCM5 is occupied by WT-DDX21 rather than MUT-DDX21. **D** ChIP-qPCR assay showing the enrichment of endogenous DDX21 at MCM5 locus in HCT116 cells. ****P < 0.0001. Results are from three biological replicates. **E** ChIP-qPCR assay showing the enrichment of exogenous WT-DDX21 and MUT-DDX21-IDR rather than MUT-DDX21 at MCM5 locus in SW480 cells. *P < 0.05, **P < 0.01, ***P < 0.001, ****P < 0.0001. Results are from three biological replicates. **F** IHC showing the positive correlation of DDX21 and MCM5 protein expression in late CRC samples. qPCR (**G**) and Western blotting (**H**) showing the mRNA and protein expression of *MCM5* is significantly down-regulated in response to DDX21 knockdown. *P < 0.05, **P < 0.01, ***P < 0.001. Results are from three biological replicates. IHC showing MCM5 protein level is positively correlated to DDX21 in CRC to liver metastatic models (**I**) and lung metastatic models (**J**). The protein expression was determined by IHC score. **K** qPCR showing MCM5 expression is positively correlated to DDX21 in DDX21 overexpressed CRC cells, and DDX21 mutations disrupt MCM5 expression, which is rescued by IDR fusion. **P < 0.01, ***P < 0.001, ****P < 0.0001. Results are from three biological replicates. **L** Western blotting showing the protein levels of MCM5 and EMT markers are down-regulated when phase separation of DDX21 is disrupted by IDR mutations, which are rescued by IDR fusion. β-actin is used as a loading control. Two biological replicates are assayed for western blotting experiment. The data in (**D**), (**E**), (**G**), (**I**), (**J**) and (**K**) are presented as the means ± SD.

To elucidate the molecular mechanism and direct targets of DDX21 phase separation on CRC metastasis, we performed RNA-seq and DDX21 ChIP-seq using different DDX21 patterns. The direct targets of DDX21 phase-separated condensates were obtained using the following standards in order: (1) collect the significant differentially expressed genes (DEGs) in response to DDX21 knockdown via RNA-seq, (2) identify the effective targets of DDX21 by collecting the overlap between (1) and WT-DDX21 targeting genes via ChIP-seq, and (3) exclude the mutant DDX21 targeting genes from (2) (Fig. 5A). Since the direct effect of mutations is to disrupt DDX21 phase separation, these standards will endow the direct targets not only for DDX21 but rather DDX21 phase separation. We collected 576 of significant differentially expressed genes in response to DDX21 knockdown, 4211 of WT-DDX21 targeting genes and 1660 of mutant DDX21 targeting genes, respectively. By these standards, we finally identified 29 genes as the targets of DDX21 phase-separated condensates (Fig. 5B). Correlation analysis between DDX21 and the 29 genes using TCGA CRC cohort showed that, among them, *MCM5, SGO2, TARS, GAS2L3* and *XXYLT1* were the top 5 genes most correlated with DDX21 (Table S2). Since MCM5 which is essential for the initiation of DNA replication has been reported to associated with cancer cell proliferation [31] and metastasis [32], we focused on the role of *MCM5* in DDX21-dependent CRC regulation.

ChIP-seq results showed that *MCM5* gene locus is occupied by WT-DDX21 rather than mutant DDX21 (Fig. 5C), which were further validated by ChIP-qPCR (Fig. 5D). Interestingly, Mut-IDR rescued the disabled function of DDX21 mutant on *MCM5* binding (Fig. 5E). Moreover, we observed a high correlation between DDX21 and MCM5 expressions in late CRC samples by IHC and mRNA analysis in all stage CRC samples (Fig. 5F, S4A–C). Knocking down of DDX21 significantly reduced MCM5 expression in CRC cell lines and CRC to liver and lung metastatic models (Fig. 5G–J, S4D), while overexpression of WT-DDX21 induced MCM5 expression (Fig. 5K, L). Notably, overexpression of mutant DDX21 attenuated MCM5 induction, which is restored by IDR fusion (Fig. 5K, L), further indicating that *MCM5* is a direct target of DDX21 phase separation. Similar results were also observed that overexpression of WT-DDX21 led to the induction of EMT markers (Fig. 5L), which can be attenuated by acidic mutations. As expected, the disabled function of DDX21 mutant on EMT expression was restored by IDR fusion (Fig. 5L), indicating that EMT pathway may be also involved in the phase separation-dependent mechanism.

Collectively, these results demonstrate that DDX21 phase-separated condensates directly target on *MCM5* to induce its expression.

### MCM5 is required for DDX21 phase separation-induced metastasis

**Fig. 6:**
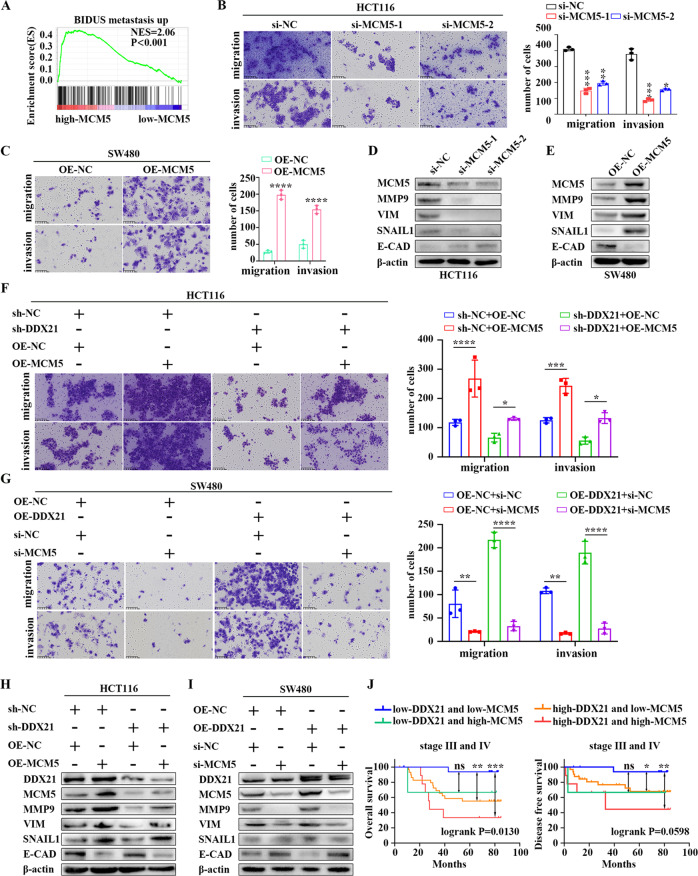
MCM5 is required for DDX21 phase separation-induced metastasis. **A** GSEA analysis based on high-MCM5 group and low-MCM5 group in TCGA CRC cohort. The migration and invasion ability of CRC cells with MCM5 knockdown (**B**) or overexpression (**C**) are analyzed by transwell assay. *P < 0.05, **P < 0.01, ***P < 0.001. Results are from three biological replicates. Protein level of EMT markers in MCM5 knockdown (**D**) or overexpression (**E**) cells are showed by Western blots. β-actin is used as a loading control. Two biological replicates are assayed for western blotting experiment. **F** Knockdown of DDX21 attenuates HCT116 cell migration and invasion, which can be rescued by overexpression of MCM5. *P < 0.05, ***P < 0.001, ****P < 0.0001. Results are from three biological replicates. **G** DDX21 overexpression promotes CRC cell migration and invasion in SW480 cells, which are abolished by knockdown of *MCM5*. **P < 0.01, ****P < 0.0001. Results are from three biological replicates. **H** Knockdown of DDX21 decreases the protein level of EMT markers in HCT116 cells, which can be rescued by overexpression of MCM5. β-actin is used as a loading control. Two biological replicates are assayed for western blotting experiment. **I** DDX21 overexpression increases the protein level of EMT markers in SW480 cells, which are abolished by knockdown of *MCM5*. β-actin is used as a loading control. Two biological replicates are assayed for western blotting experiment. **J** Kaplan-Meier analysis of the OS rate and DFS rate in CRC patients of stage III and IV based on the combined DDX21 and MCM5 expression. **P < 0.01, ***P < 0.001. The data in (**B**), (**C**), (**F**) and (**G**) are presented as the means ± SD.

To determine whether MCM5 participates in the metastatic function of DDX21 phase separation, we first identified the role of MCM5 in CRC. We found high MCM5 group was also significantly enriched in “BIDUS metastasis up” gene set from TCGA database (Fig. 6A). To verify this, we knocked down *MCM5* in HCT116 cell line while overexpressed *MCM5* in SW480 cell line (Fig. S5A, B). Results showed that, as compared to control cells, knockdown of *MCM5* dramatically suppressed migration and invasion abilities of HCT116 cells, whereas overexpression of *MCM5* promoted these abilities of SW480 cells (Fig. 6B, C). Also, our result revealed that downregulation of MCM5 remarkedly suppressed the proliferation of HCT116 cells and overexpression of MCM5 enhanced the proliferation of SW480 cells (Fig. S5C, D). Moreover, GSEA enrichment analysis revealed that MCM5 expression was significantly correlated with “HALLMARK EMT” and “KEGG focal adhesion” gene sets (Fig. S5E, F). In line with this, the expression of EMT markers were down-regulated in response to knockdown of *MCM5*, whereas up-regulated in response to *MCM5* overexpression (Fig. 6D, E). These results reveal that MCM5 is important for CRC metastasis.

Next, we asked whether MCM5 is necessary for DDX21 phase separation-dependent CRC metastasis. We found that knockdown of *DDX21* significantly reduced the migration and invasion ability of CRC cells as well as the expressions of EMT markers, both of which can be rescued by overexpression of *MCM5* (Fig. 6F, H). Similarly, overexpression of *DDX21* significantly enhanced the migration and invasion ability of CRC cells as well as the expressions of EMT markers, both of which can be abolished by knockdown of *MCM5* (Fig. 6G, I). Additionally, rescue assays indicated that MCM5 contributed to DDX21-induced proliferation in vitro and in vivo (Fig. S5G–K). Collectively, these results indicate that MCM5 is required for DDX21-induced metastasis of CRC.

Finally, we investigated the clinical significance of DDX21 and MCM5 in CRC. Consistent with DDX21, high MCM5 expression predicts poor OS rate and DFS rate in stage III and IV CRC patients (Fig. S6A, D), rather than among all stage or stage I and II (Fig. S6B, C, E, F). Since both of DDX21 and MCM5 are important for CRC metastasis, we asked the OS rate of both high-expressing group. As expected, in stage III and IV CRC patients, individuals with high DDX21 and high MCM5 expression had the poorest OS and DFS (Fig. 6J), rather than among all stage or stage I and II (Fig. S6G–J). Thus, a synergistic higher expression of DDX21 and MCM5 can be used as a biomarker for CRC with high malignance.

**Fig. 7:**
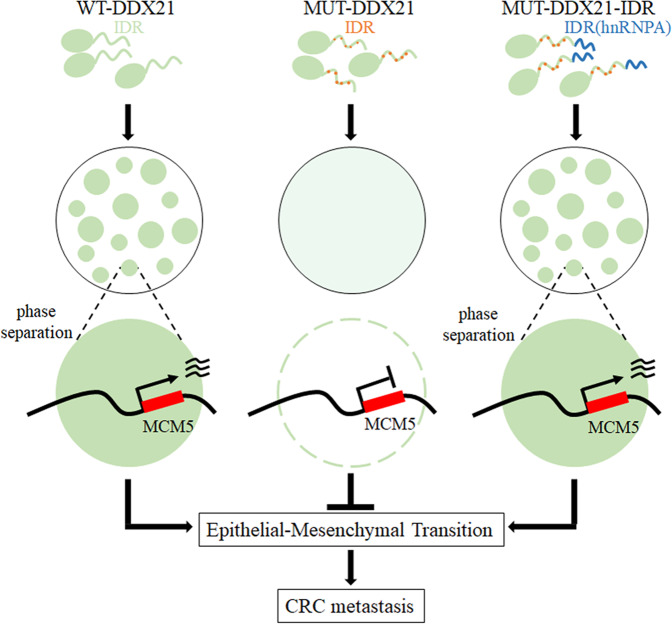
Schematic illustration showing the underlaying molecular mechanism of DDX21/phase separation/MCM5 axis-mediated CRC metastasis. In brief, phase separation of DDX21 promotes colorectal cancer metastasis via MCM5-dependent EMT pathway, while acidic mutations abolish DDX21 phase separation and hnRNPA1-IDR fusion recover the phase separated ability of the mutant.

Altogether, the phase separated condensates of DDX21 target and activate MCM5, which further initiates the activation of EMT pathway for late stage CRC metastasis. Therefore, our results provide a now model how RBPs regulate cancer metastasis through phase separation (Fig. 7).

## Discussion

At present, several types of mutations have been designed at IDR to specific disrupt phase separation capacity of proteins. Acid mutation in which glutamic acid and aspartic acid is usually replaced with alanine (E/D to A) remarkably abrogate phase separation induced by abolishing the ionic interaction [6, 10, 33]. Serine mutation in which all serine residues are replaced with alanine (S to A) can also disrupt phase separation of coactivators such as MED1 [7]. Aromatic mutation is another mutation type in which aromatic amino acids such as tyrosine, phenylalanine and tryptophan are replaced with alanine (F/Y/W to A). This type of mutation can attenuate cation-π interactions [34]. Since glutamic and aspartic acids are enriched at the IDR of DDX21, we employed acidic mutation to intervene its phase separation ability. Although these kinds of mutations can effectively impact phase separation, questions are asked that whether these large scale mutations influence other functions of proteins beside phase separation. Therefore, a reliable algorithm needs to be developed to exactly identify the key amino acid residues for phase separation. The predicted residues can be selected as the mutation sites to disrupt phase separation without influence other functions of protein.

RBPs which have both RRM and IDR are likely to phase separated [33]. Therefore, we compared the structure difference among different types of RBPs. hnRNPA1 is comprised of two folded RRMs and one IDR [14]. Similar with hnRNPA1, TDP43 contains two folded RRM, one folded N-terminal domain (NTD) and one IDR at C-terminal. The C-terminal IDR which harbors a highly aggregation-prone domain contributes most to its phase separation [35]. FUS is another RBPs consisting of an extended IDR, including the SYGQ-rich domain at N-terminal and RGG domains at C-terminal. The RGG/RG motifs that are enriched in its IDR are critically contributing to FUS phase separation [36]. Another well characterized RBP, Tau, contains a NTD, a proline-rich domain (PRD), a microtubule-binding domain (MTBD) and a C-terminal domain (CTD). Tau also undergoes phase separation by the interaction of N‐terminal half and C‐terminal MTBD [37]. As a DDX family member, DDX4 is comprised of a DEAD-box RNA helicase domain in the central, an extended NTD and CTD, and the disordered NTD facilitates its phase-separated droplets behavior [38]. Consistent with DDX4, we found that DDX21 contains an IDR at its N-terminal, a DEAD-box RNA helicase domain in the central, and an extended CTD. We found the N-terminal IDR of DDX family proteins contributes most to their phase separation ability. Since these DDX-specific IDRs are enriched with acidic amino acids, replacement of these amino acids notably disrupt their phase separation ability. Since RRMs are also functional domains contributing to phase separation [14], future work will be focused on seeking new strategies to intervene phase separation on these domains.

According to our GSEA analysis result, P53 pathway, KRAS signaling pathway, oxidative phosphorylation and EMT were involved in DDX21-regualted CRC progression. Recently, Katarzyna et al. have been reported that P53 pathway was one of the pathways that regulated by DDX21. They also confirmed that loss of DDX21 in human ECs remarkedly upregulated the P53 and P21 expression [39]. Also, KRAS has been reported as a frequently mutated proto-oncogene in CRC and KRAS signaling pathway plays an important role in tumorigenesis [40]. The effect of KRAS signaling pathway works mainly through its downstream signaling pathways, such as PI3K/AKT/mTOR, MAPK/ERK, RAF/MEK/ERK and RALGEF/RAL [41]. Oxidative phosphorylation is an important intracellular metabolic process, which has a significant impact on the response to anticancer therapy via inducing cancer resistance [42]. Our results showed that loss of DDX21 affected the P53 pathway and KRAS signaling pathway (data not shown).

In our previous study, we focused on the molecular mechanism of CRC metastasis. However, the study of DDX21 in CRC metastasis has not been reported so far. EMT has been implicated in cancer metastasis for decades, which refers to the biological process of epithelial cells transforming into mesenchymal phenotype cells through specific procedures and plays a fundamental role in cancer progression to metastasis or diseases like organ fibrosis [43, 44]. Accumulating studies show that EMT contributes to CRC metastasis, progression and drug resistance. EMT markers (such as depletion of E-cadherin expression and overexpression of vimentin) might serve as potential therapeutic targets and predict survival outcome of CRC patients [44]. During EMT, epithelial cells lose epithelial phenotypes such as cell polarity and connection with the basement membrane, and obtain mesenchymal phenotypes such as higher migration and invasion and the ability to degrade extracellular matrix [45]. Since DEAD/H-BOX family has the potential to form phase separation, the member DDX21 may also undergo phase separation in CRC. However, little is known about the underlying mechanism between phase separation and EMT in CRC. Our study revealed that DDX21 forms phase-separated condensates with liquid-like behavior in CRC and further promotes CRC cell metastasis via EMT process.

ChIP-seq is a useful tool to identify the genome targets of proteins, such as transcription factors [46–48]. However, few studies identify phase separation-specific targets till now. Here, we compared the genome targets of WT-DDX21 and MUT-DDX21, considered expressions meanwhile, to identify phase separation-specific targeting genes. The method is groundbreaking, as we can only block the targets to suppress cancer phenotype without impacting phase separation. This hypothesis is validated by our study that, since MCM5 is a phase separation-specific target of DDX21, we can suppress cancer metastasis by inhibiting MCM5 expression without disrupting DDX21 phase separation. As phase separation covers large scale of genome and disrupting phase separation may influence normal gene expression, this design may attenuate side effect to some extent.

## Materials and methods

### Samples and cell lines

Paraffin tissues of CRC were collected from the Department of Pathology of the First Affiliated Hospital of Sun Yat-Sen University in 2013 and made into tissue microarray. There were totally 207 cases of paired cancer and adjacent tissues. This study was approved by the informed consent of patients and the institutional research ethics committee. Two pathologists identified the histopathological features of CRC according to WHO criteria. Human CRC cells HT29, HCT116, LOVO, RKO, SW480 and SW620 were purchased from national infrastructure of cell line resource, China and normal colonic epithelial cells NCM460 were purchased from Guangzhou Xinyuan technology company, China. Cells were culture under their guidance.

### Migration and invasion assay

The transwell assay and wound healing assays were performed as described previously [49].

### Cell proliferation assay

Cell proliferation assays were performed by a cell counting kit-8(CCK8) (Bimake #B34304, China) according to the manufacturer’s guideline. Briefly, 1 × 10^3^ cells were seeded into a 96-well plate, and the absorbance values of each well were measured every day until 5 days.

### Animal experiments

All 4–6 weeks old of female Balb/c nude mice were purchased from the GemPharmtech. For liver metastasis assay, after anesthetized, the left upper abdomen of nude mice was incised about 1.0 cm, and the spleen was gently pulled out from the abdominal cavity with tissue tweezers. Then 1 × 10^6^ cells/mice were slowly injected into the spleen of nude mice (*n* = 5 per group). After the injection, the needle eye was compressed with 75% alcohol cotton ball for 2–3 min to stop bleeding, and the incision of muscle layer and skin was sutured. The mice were sacrificed after intrasplenic injection for 6–7 weeks. The mouse liver was collected to check the metastatic rate. For lung metastasis assay, 2 × 10^6^ cells/mice were injected to the tail vein of nude mice (*n* = 6 per group). After 2 months, the heart, lung and liver of nude mice were collected to observe the metastatic rate. For xenograft assay, 1 × 10^7^ cells/mice were subcutaneously injected into the right armpit of nude mice (*n* = 6 per group). Tumor volume were measured every 3 days for around 15 days. These experiments were approved by the Animal Ethics Committee of Sun Yat-Sen University.

### Chromatin immunoprecipitation (ChIP)

ChIP assay performed with a ChIP kit (Beyotime, #P2078, China) according to manufacturer’s instruction. Briefly, HCT116 cells and SW480-MUT-DDX21 cells were crosslinked with 1% formaldehyde for 10 min at room temperature and lysed with SDS lysis buffer. DNA was fragmented to 200–500 bp by ultrasound, and the lysate was immunoprecipitated with protein A/G magnetic beads (Biolinkedin, #L-1004, China) and IgG control antibody or DDX21 antibody of ChIP-grade. After purified, DNA was applied for sequencing and qPCR. The primer sequences for ChIP-qPCR are listed in Table S1.

### RNA-seq analysis

The RNA of three HCT116-si-NC and three HCT116-si-DDX21 cell lines were isolated for RNA-seq. All RNA samples meet a criterion to high quality. Differential expression analysis of HCT116-si-NC and HCT116-si-DDX21 was performed by the DESeq R package (1.18.0). According to the negative binomial distribution, DESeq provided statistical routines to determine differential expression in digital gene expression data by using a model. The resulting P-values were adjusted through the Benjamini and Hochberg’s method for controlling the false discovery rate. Genes with an adjusted P-value <0.05 were regarded as differentially expressed.

### siRNA, Plasmid and lentivirus preparation and infection

The sequences of used siRNAs and Plasmids were listed in Table S1. Lentiviruses were produced by using Lipofectamine 3000 (Invitrogen, #2270665, CA) to co-transfect the above plasmids with two packaging plasmids (Δ8.0 and VSVG) into HEK293T cells for 48 h. To obtain stable transfection CRC cell lines, CRC cells were infected with the lentivirus with 2 μg/mL polybrene. Then, the cells were screened for subsequent experiments under a concentration of 2 or 4 mg/mL puromycin.

### Protein expression and purification

Plasmids containing His × 6-tagged were transformed into E.coli BL21 (DE3) cells. Then bacteria were incubated into LB medium containing 1 mg/mL kanamycin and grown overnight at room temperature. The bacteria solution was expanded to 500 mL LB medium with kanamycin, and after growing for 5 h until the OD600 value of bacteria solution reached 0.6–0.8. Then the expression of proteins was induced by adding 0.3 mM IPTG into the solution at 16 °C. Protein purification was completed by Protein Purification Kit (Beyotime, #P2226, China) based on the manufacturer’s guidelines.

### Immunofluorescence (IF) staining

Cells were seeded into glass coverslips in 24-well plates and cultured for 48 h and then cells were fixed with 4% paraformaldehyde, blocked by 10% FBS. Subsequently, cells were incubated with the primary antibody DDX21 overnight at 4 °C. After incubation with an Alex-Fluor-488-conjugated secondary antibody for 30 min at room temperature and counterstained with DAPI. Finally, the fixed cells were imaged using N-SIM or confocal microscopy.

### In vitro droplet formation assay

Varying concentrations of purified proteins were added to in the buffer containing 10% PEG8000, 125–500 mM NaCl and water. Then loaded the protein solution (10 μL) onto a glass slide immediately with a coverslip, and imaged by microscope (Nikon).

### Fluorescence recovery after photobleaching (FRAP)

FRAP experiments were performed on Nikon Eclipse Ti with 488 nm laser. Images were obtained by the NIS-Elements software. Briefly, photobleaching was performed in the central region of droplets using the 488 nm laser line. Fluorescence recovery was monitored every 1 s for 60 s immediately after photobleaching. The fluorescence intensity and the images of the bleached region, reference region and background region were recorded by the FRAP module in the ZEN software. The data were analyzed with Prism 8 (Graphpad Software, La Jolla, CA).

### Quantitative real-time PCR (qPCR), Western blotting and Immunohistochemical (IHC) staining

qPCR, Western blotting and IHC staining were performed as previously described [49]. Relative primers used for qPCR and the antibodies used for Western blotting and IHC staining are shown in Table S1.

### Bioinformatics analysis

The gene expression data of TCGA were downloaded from UCSC website [26], including 380 cases of CRC tissues and 51 cases of corresponding adjacent tissues. The CRC expression data of GSE21510 [27] and GSE32323 [28] were downloaded from GEO database. Among them, GSE21510 included 19 cases of CRC tissues and 25 cases of corresponding adjacent tissues, GSE32323 included 17 cases of CRC tissues and 17 cases of corresponding adjacent tissues. The IDR regions of DDX21 were predicted through the IUPred2A online tool (https://iupred2a.elte.hu/plot) [50]. Correlation coefficients in Fig. S4 was downloaded from GEPIA2 (http://gepia2.cancer-pku.cn/#index) according to TCGA COAD and READ cohorts.

### Statistics

Statistical analysis was performed by SPSS 25.0 and GraphPad Prism 8. The data between two groups were compared by unpaired T test. Different parameters were analyzed by Chi-square tests and Fisher’s exact test. Pearman correlation analysis was used to evaluate the correlation between two variables. Kaplan-Meier method was used to analyze the survival rate of CRC patients with high and low DDX21 expression or MCM5 expression. All data were presented as mean ± SD. All experiments were performed at least 3 times. *P* < 0.05 was considered statistically significant.

## Supplementary information

supplematary figure legends

supplementary figure 1

supplematary figure 2

supplematary figure 3

supplematary figure 4

supplematary figure 5

supplematary figure 6

supplementary table s1

supplementary table s2

## Data Availability

The original contributions presented in the study are included in the article/supplementary material. Further inquiries can be directed to the corresponding authors.
