# Supplementary material for: Phase separation of DDX21 promotes colorectal cancer metastasis via MCM5-dependent EMT pathway
Source: Oncogene. 2023 Apr 7;42(21):1704–15. doi: 10.1038/s41388-023-02687-6 (PMC10202810; doi:10.1038/s41388-023-02687-6)
Supplement: Supplementary file 1 — supplematary figure legends [file 41388_2023_2687_MOESM1_ESM.docx]

**Phase separation of DDX21 promotes colorectal cancer metastasis via MCM5-dependent EMT pathway**

**Supplement figure 1**

(A-C) Heatmap show DEGs of DEAD/H family in CRC based on TCGA CRC cohort (A), GSE21510 (B) and GSE32323 (C). (D) Western blots show that DDX21 expression in NCM460 and CRC cell lines. β-actin is used as a loading control. Two biological replicates are assayed for western blotting experiment. (E-H) correlation between DDX21 expression and OS and DFS in all stage and stage I and II of CRC. (I and J) the mRNA and protein level of DDX21 expression were evaluated in DDX21 knockdown and overexpressed CRC cells by qPCR and Western blot assays. ****P*<0.001, *****P*<0.0001. Results are from three biological replicates. (K and L) Wound healing assay was performed to revealed the migrated ability of DDX21. *****P*<0.0001. Results are from three biological replicates. (M and N) The influence of DDX21 knockdown on HCT116 (M) or RKO (N) cell proliferation ability is evaluated by CCK8 assay. ****P<0.0001. (O) The influence of DDX21 overexpression on SW480 cell proliferation ability is evaluated by CCK8 assay. ****P<0.0001. (P) Western blot results showed DDX21 expression in HCT116 decreased significantly by stable interference. β-actin is used as a loading control. Two biological replicates are assayed for western blotting experiment. (Q and R) Representative images of cardiac metastasis and soft tissue metastasis was derived from sh-NC and sh-DDX21 group of lung metastasis model. Metastatic incidence of cardiac metastasis and soft tissue metastasis was calculated. The data in (I), (J) and (L) are presented as the means ± SD.

**Supplement figure 2**

(A and B) EMT (Hallmark gene set) and KEGG focal adhesion gene sets enriched in high-DDX21 vs low-DDX21 group in TCGA CRC cohort. (C and D) Representative IHC images of DDX21, E-cadherin and vimentin staining in 2 cancerous nodes are derived from soft tissue metastasis of HCT116-sh-NC group. The protein expression was determined by IHC score. The data in (D) are presented as the means ± SD.

**Supplement figure 3**

(A) The protein sequences of the MUT-DDX21 protein. (B) Representative images and quantitative analysis of droplet formation of MUT-IDR at the indicated protein concentrations in droplet formation buffer with 125 mM NaCl and 10% PEG. The number of droplets increases with increased protein concentration. **P*<0.05, *****P*<0.0001. (C) The transfect efficiency of GFP, WT-DDX21, acidic mutant DDX21 and Mut-IDR was analyzed by qPCR. ****P*<0.001, *****P*<0.0001. Results are from three biological replicates. (D) Wound healing assay is used to demonstrate the migrated ability of HT29-GFP, WT-DDX21, MUT-DDX21 and MUT-IDR cells. ***P*<0.01, ****P*<0.001. Results are from three biological replicates. (H) Migration and invasion ability of the above cells were analyzed by transwell assay. **P*<0.05, ***P*<0.01, ****P*<0.001. Results are from three biological replicates. The data in (B-E) are presented as the means ± SD.

**Supplement figure 4**

(A-C) correlation between DDX21 mRNA and MCM5 mRNA in TCGA cohort (A), GSE21510 cohort (B) and GSE32323 cohort (C). (D) Western blots show that MCM5 expression is significantly downregulated in DDX21 stable knockdown cells compared to control cells. β-actin is used as a loading control. Two biological replicates are assayed for western blotting experiment.

**Supplement figure 5**

(A and B) The expression of MCM5 was evaluated in MCM5 knockdown and overexpressed CRC cells by qPCR assay. ****P*<0.001, *****P*<0.0001. Results are from three biological replicates. (C) The influence of MCM5 knockdown on HCT116 cell proliferation ability is evaluated by CCK8 assay. ****P<0.0001. (D) The influence of MCM5 overexpression on SW480 cell proliferation ability is evaluated by CCK8 assay. ****P<0.0001. (E and F) EMT (Hallmark gene set) and KEGG focal adhesion gene sets enriched in low-MCM5 group. (G) Knockdown of DDX21 attenuates HCT116 cell proliferation, which can be rescued by overexpression of MCM5. ****P<0.0001. (H) DDX21 overexpression promotes CRC cell proliferation in SW480 cells, which are abolished by knockdown of MCM5. ****P<0.0001. (I) Representative images of CRC tumor derived from sh-NC+OE-NC, sh-NC+OE-MCM5, sh-DDX21+OE-NC and sh-DDX21+OE-MCM5 group of nude mice. *P<0.05. (J and K) tumor volume and tumor weight of the above group are shown. *P<0.05, **P<0.01, ****P<0.0001. The data in (A), (B) and (K) are presented as the means ± SD.

**Supplement figure 6**

(A-C) Kaplan-Meier analysis of the OS rate in CRC patients of stage III and IV (A), all stage (B) and stage I and II (C) according to MCM5 expression. (D-F) Kaplan-Meier analysis of the DFS rate in CRC patients of stage III and IV (D), all stage (E) and stage I and II (F) according to MCM5 expression. (G and H) Kaplan-Meier analysis of the OS rate in CRC patients of all stage (G) and stage I and II (H) according to combined DDX21 and MCM5 expression. (I and J) Kaplan-Meier analysis of the DFS rate in CRC patients of all stage (I) and stage I and II (J) according to combined DDX21 and MCM5 expression.
