# Supplementary figures and images for: Phase separation of DDX21 promotes colorectal cancer metastasis via MCM5-dependent EMT pathway
Source: Oncogene. 2023 Apr 7;42(21):1704–15. doi: 10.1038/s41388-023-02687-6 (PMC10202810; doi:10.1038/s41388-023-02687-6)

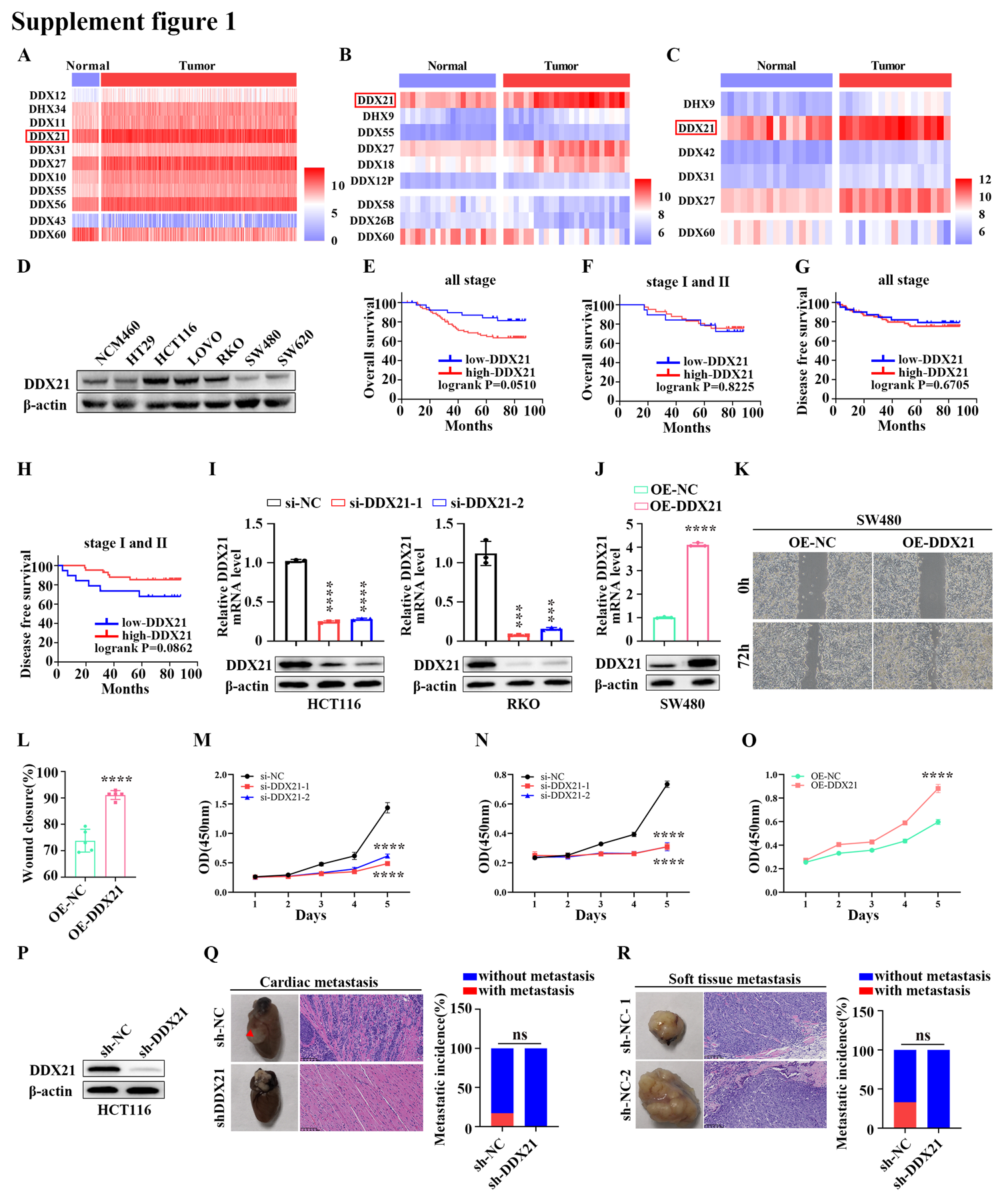

Supplement: Supplementary file 2 — supplementary figure 1 [file 41388_2023_2687_MOESM2_ESM.tif]

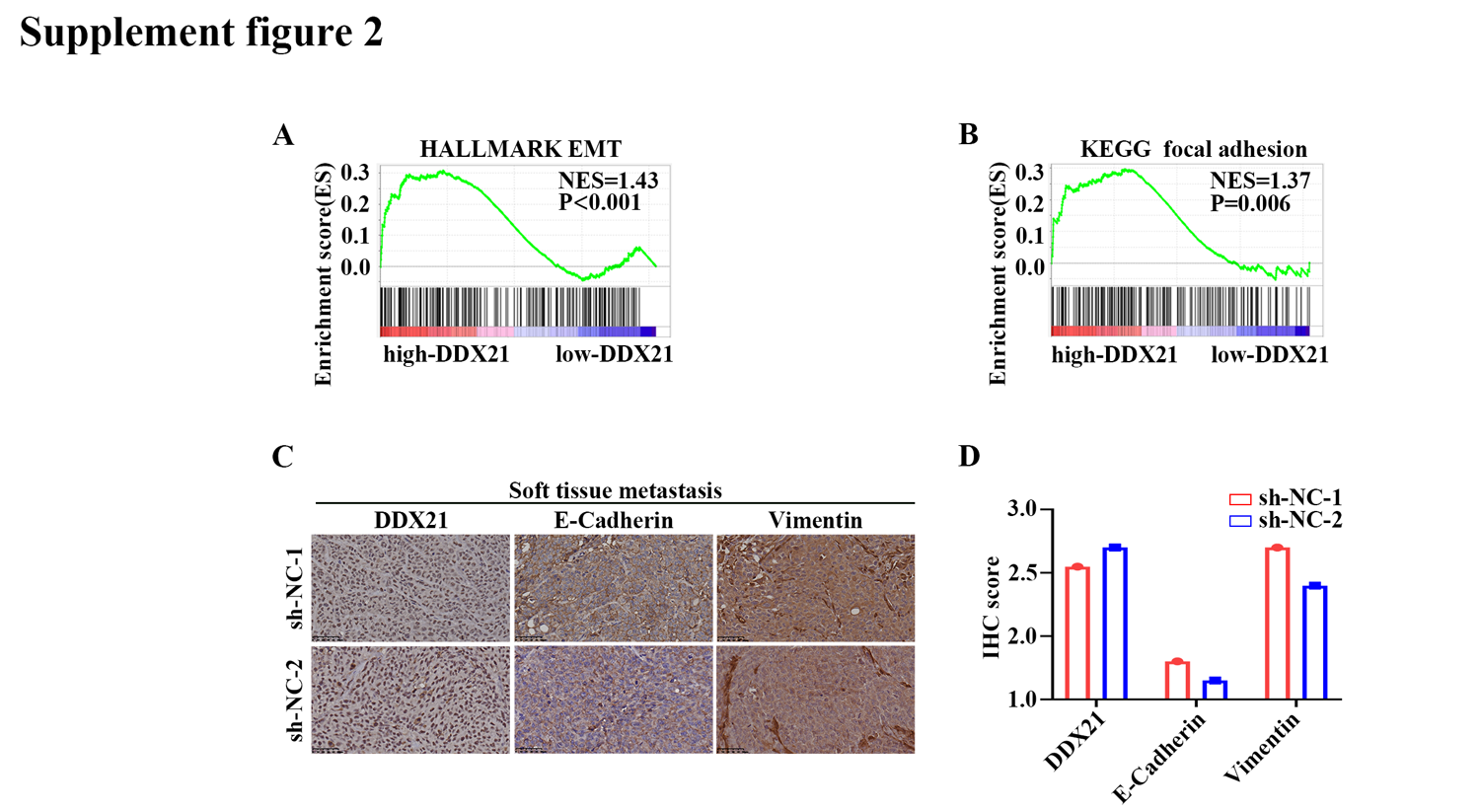

Supplement: Supplementary file 3 — supplematary figure 2 [file 41388_2023_2687_MOESM3_ESM.tif]

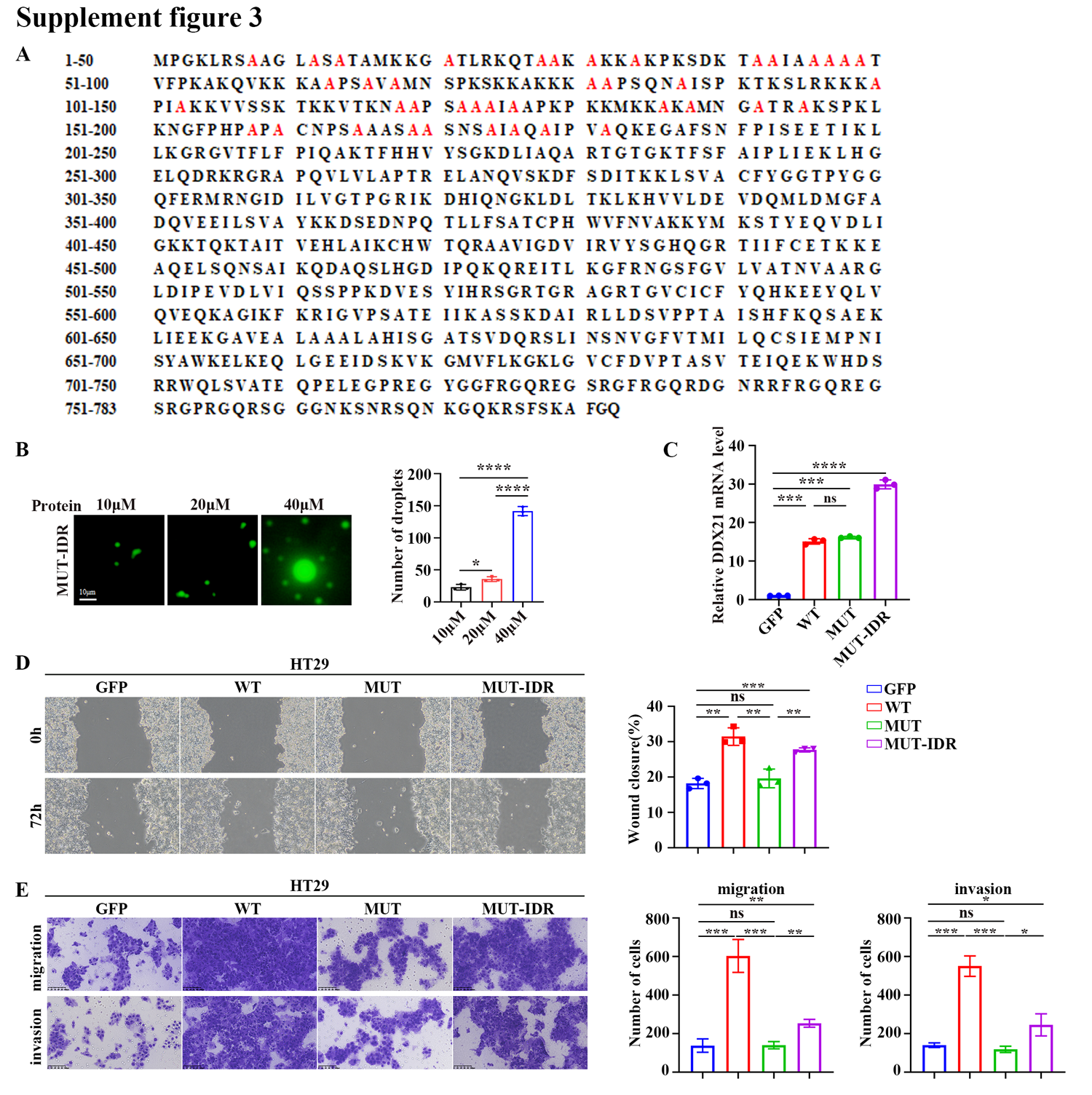

Supplement: Supplementary file 4 — supplematary figure 3 [file 41388_2023_2687_MOESM4_ESM.tif]

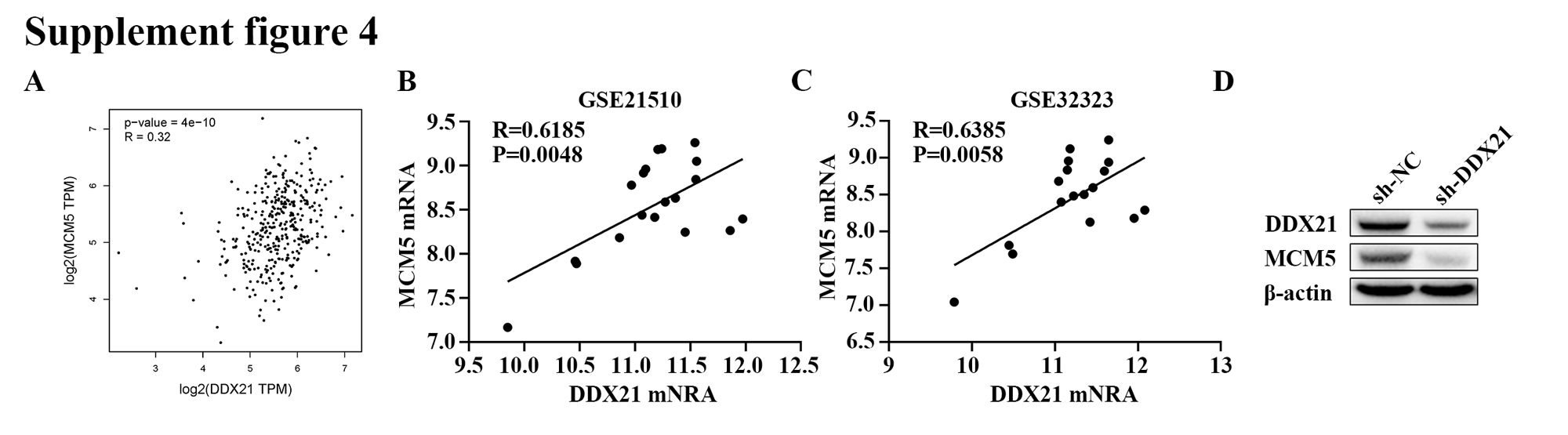

Supplement: Supplementary file 5 — supplematary figure 4 [file 41388_2023_2687_MOESM5_ESM.tif]

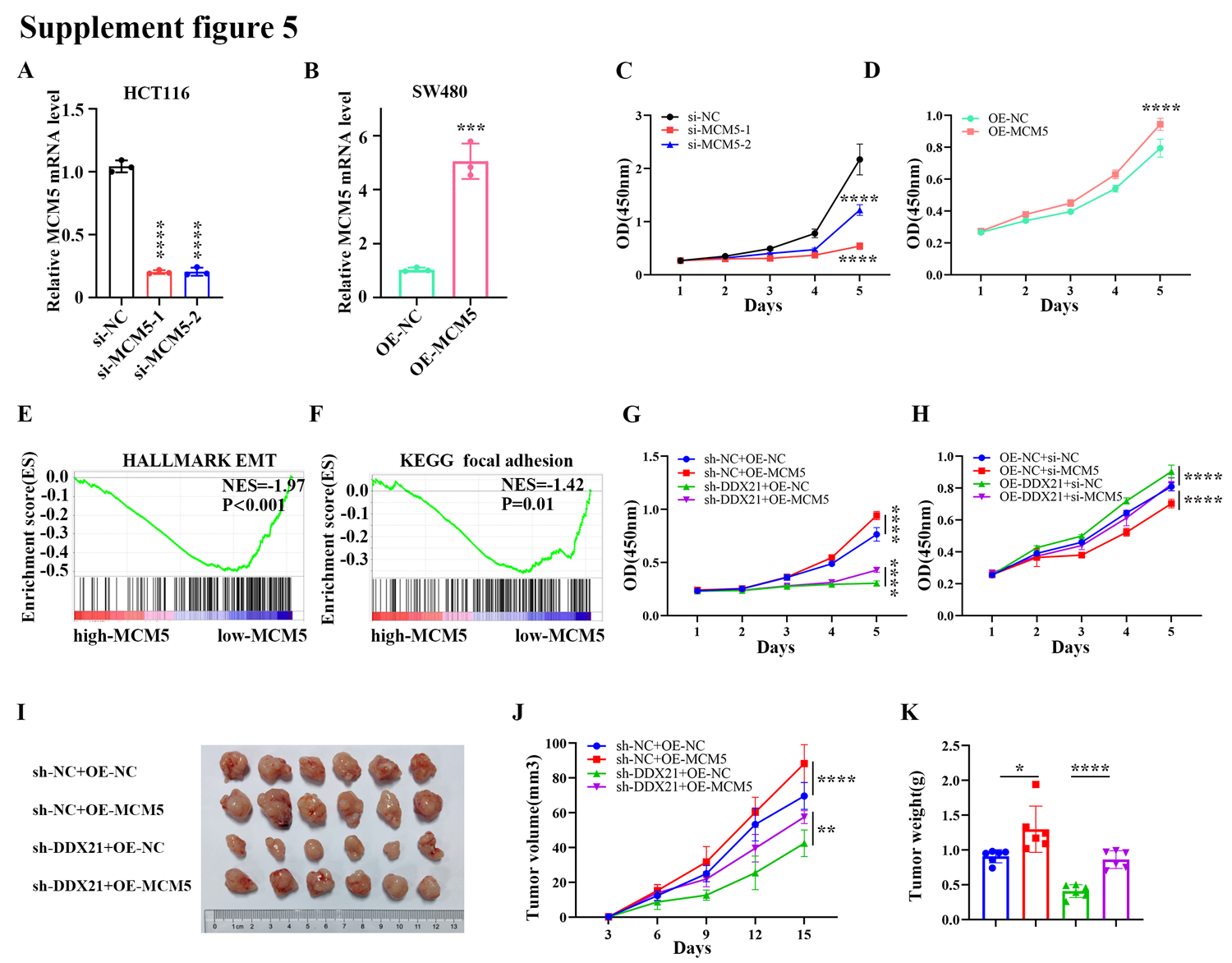

Supplement: Supplementary file 6 — supplematary figure 5 [file 41388_2023_2687_MOESM6_ESM.tif]

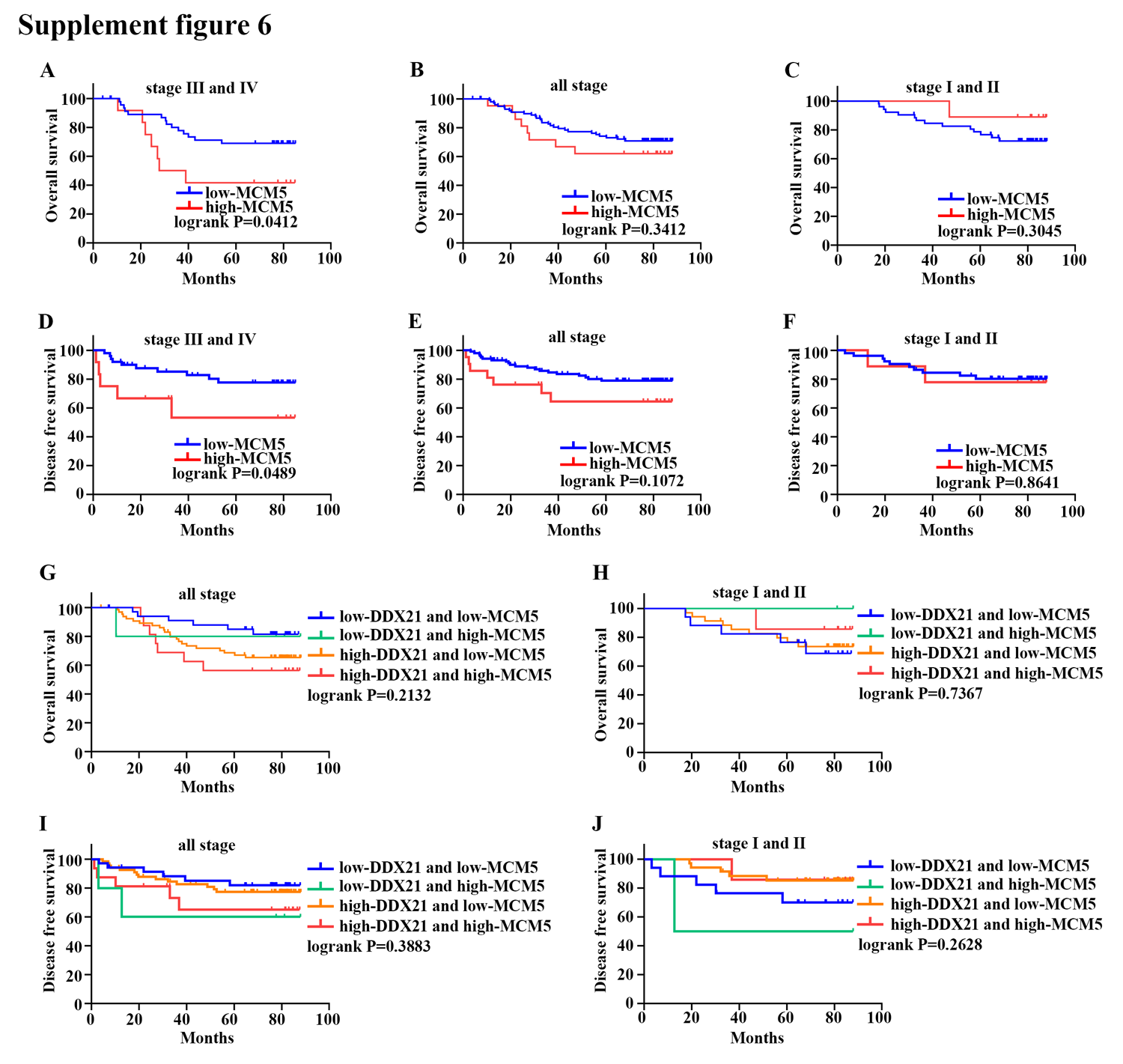

Supplement: Supplementary file 7 — supplematary figure 6 [file 41388_2023_2687_MOESM7_ESM.tif]
