# Supplementary material for: Phase separation of DDX21 promotes colorectal cancer metastasis via MCM5-dependent EMT pathway
Source: Oncogene. 2023 Apr 7;42(21):1704–15. doi: 10.1038/s41388-023-02687-6 (PMC10202810; doi:10.1038/s41388-023-02687-6)
Supplement: Supplementary file 8 — supplementary table s1 [file 41388_2023_2687_MOESM8_ESM.docx]

| **Table S1 Sequences of all primers, antibodies, and plasmids in our study** | | |
| --- | --- | --- |
| **Primers sequence** |  |  |
| β-actin-F | CCTGGCACCCAGCACAAT |  |
| β-actin-R | GGGCCGGACTCGTCATAC |  |
| DDX21-F | TCATCAAGGACGCACTATCATCT |  |
| DDX21-R | CCTTTCAGGGTGATTTCCCTTT |  |
| MCM5-F | GAAGATCCCTGGCATCATCATC |  |
| MCM5-R | ATTTGTCGGGCATGATGAAGTA |  |
| MCM5-CHIP-F | TAGGAAGGGGTGTCTGGAGA |  |
| MCM5-CHIP-R | TCTATCGCCGTCTCCAACTC |  |
|  |  |  |
| si-DDX21-1 | GGAAATGAATGGAGAAACT |  |
| si-DDX21-2 | GGAATTAAGTTCAAACGAA |  |
| si-MCM5-1 | GCATCTACTCCATCAAGAA |  |
| si-MCM5-2 | GCTCCCTGATGGACTTACT |  |
| sh-DDX21 | GCATGAGGAATGGGATTGATA |  |
|  |  |  |
| **Category** | **Source** | **Cat. No.** |
| Antibodies |  |  |
| DDX21 Polyclonal antibody | proteintech | #10528-1-AP |
| MCM5 Polyclonal antibody | proteintech | #11703-1-AP |
| Beta Actin Polyclonal antibody | proteintech | #20536-1-AP |
| E-Cadherin (24E10) Rabbit mAb | cell signaling technology | #3195 |
| Vimentin (D21H3) XP® Rabbit mAb | cell signaling technology | #5741 |
| MMP-9 (D6O3H) XP® Rabbit mAb | cell signaling technology | #13667 |
| Snail (C15D3) Rabbit mAb | cell signaling technology | #3879 |
| Anti-mouse IgG (H+L), F(ab')2 Fragment (Alexa Fluor® 488 Conjugate) | cell signaling technology | #4408 |
| DAPI | cell signaling technology | #4083 |
| Normal Rabbit IgG | cell signaling technology | #2729 |
|  |  |  |
| **Plasmids** |  |  |
| pLKO.1-puro-scramble | IGEbio |  |
| pLKO.1-puro-sh-DDX21 | IGEbio |  |
| pCDH-CMV-MCS-EF1-puro | IGEbio |  |
| pCDH-CMV-flag-DDX21-EF1-puro | IGEbio |  |
| pCDH-CMV-MCS-EGFP-EF1-puro | IGEbio |  |
| pCDH-CMV-EGFP-DDX21-WT-EF1-puro | IGEbio |  |
| pCDH-CMV-EGFP-DDX21-MUT-EF1-puro | IGEbio |  |
| pCDH-CMV-EGFP-DDX21-MUT-IDR-EF1-puro | IGEbio |  |
| pCDH-CMV-EGFP-MCM5-EF1-puro | IGEbio |  |
| pet28a-EGFP-DDX21-WT | IGEbio |  |
| pet28a-EGFP-DDX21-MUT | IGEbio |  |
| pet28a-EGFP-DDX21-MUT-IDR | IGEbio |  |
