# Supplementary material for: Phase separation of DDX21 promotes colorectal cancer metastasis via MCM5-dependent EMT pathway
Source: Oncogene. 2023 Apr 7;42(21):1704–15. doi: 10.1038/s41388-023-02687-6 (PMC10202810; doi:10.1038/s41388-023-02687-6)
Supplement: Supplementary file 9 — supplementary table s2 [file 41388_2023_2687_MOESM9_ESM.docx]

| **Table S2 The correlation of DDX21 and phase separation specific target genes in TCGA CRC cohort** | | |
| --- | --- | --- |
| **gene name** | **pearson R** | **P value** |
| SGO2 | 0.6 | 0 |
| TARS | 0.43 | 0 |
| GAS2L3 | 0.42 | 0 |
| MCM5 | 0.32 | 4.0*e-10 |
| XXYLT1 | 0.32 | 2.5*e-10 |
| CARS | 0.27 | 4.3*e-07 |
| SLC7A11 | 0.22 | 3.2*e-05 |
| TREML2 | 0.18 | 0.00072 |
| COL4A1 | 0.18 | 0.00069 |
| FAM107B | 0.12 | 0.019 |
| ZNF423 | 0.12 | 0.017 |
| KCTD15 | 0.12 | 0.022 |
| TIGAR | 0.11 | 0.03 |
| FZD1 | 0.082 | 0.12 |
| HRH2 | 0.075 | 0.15 |
| CMBL | 0.064 | 0.22 |
| MYEOV | 0.036 | 0.49 |
| PPEF1 | 0.022 | 0.68 |
| OR10H1 | 0.019 | 0.71 |
| ANGPT4 | -0.024 | 0.65 |
| SLC4A5 | -0.025 | 0.63 |
| RBPJL | -0.026 | 0.61 |
| ABCC3 | -0.028 | 0.59 |
| NTRK2 | -0.038 | 0.46 |
| MYO15A | -0.047 | 0.36 |
| EPS8L2 | -0.063 | 0.23 |
| GIPR | -0.083 | 0.11 |
| GNMT | -0.091 | 0.082 |
| SYT8 | -0.11 | 0.034 |
